# Supplementary material for: Diagnosis of colorectal cancer using residual transformer with mixed attention and explainable AI
Source: PLoS One. 2025 Nov 3;20(11):e0335418. doi: 10.1371/journal.pone.0335418 (PMC12582454; doi:10.1371/journal.pone.0335418)
Supplement: Attachment — Submitted filename: Response to Reviewers-Final.pdf [file pone.0335418.s002.pdf]

Point-by-point Response to Editor and Reviewers' Comments

Title: Early Diagnosis of Colorectal Cancer Using Residual Transformer with Mixed Attention and Explainable AI

Authors: *Poonam Sharma, Bhisham Sharma, Ajit Noonia, Dhirendra Prasad Yadav, Panos Liatsis*

Response to the Reviewers Comments:

Reviewer #1:

Comment 1: Lack of Clear Novelty. While the manuscript introduces a "residual transformer with mixed attention," it does not sufficiently differentiate this model from existing Vision Transformer (ViT) architectures. The conceptual approach, using transformers with GradCAM for histopathology classification, has already been reported in prior studies (Colon Cancer Detection using Vision Transformers and Explainable AI), including work that closely parallels the structure and aims of your paper. Without a clearly defined architectural innovation or substantial methodological advancement, the manuscript falls short of meeting the novelty threshold expected for publication.

Response: Thank you for your concern related to the novelty of the proposed model. Previous efforts [11] attempted to tackle this challenge using a vision transformer model, which incorporates multi-head self-attention (MHSA) to provide for global correlations of the spatial features. However, the computational costs of the MHSA are quadratic in time, and require a high volume of training data for appropriate model generalization. Moreover, the classical transformer model lacks local spatial features; consequently, local and complex textural features are not adequately captured. Further, their model was tested on the binary classification task.

The proposed Residual Next Transformer Network (RNTNet) uses ResNeXt to extract high dimensional local spatial features from colon cancer images. Further, mixed attention-based transformer encoder is applied to the flattened features obtained from the ResNeXt block, which provides local and global attention to the feature map through the overlapping block attention and grid block attention, respectively. Due to this, the model is capable of focusing on the edge and boundary regions of the colon cancer regions. In addition, the computational costs of the mixed attention block are O (M^2D^2), where M is the feature map size and D is the dimension, while those of MHSA in the classical ViT are O (M^4D), which is much higher than the proposed model. Further, we tested RNTNet on the diverse datasets KvasirV1 and Kaither, which contain 8 types of diseases, i.e., multi-class classification.

The contributions of this research are as follows:

(1) We utilized the ResNeXt, a group convolution-based residual network for high-dimensional spatial feature extraction and the ViT encoder to provide global contextual information for precise CRC diagnosis.

(2) Further, in the ViT encoder, we integrated overlapping block attention and grid attention to effectively capture local and global spatial and contextual information of the colorectal cancer regions.
(3) A Grad-Cam module was incorporated in the proposed system to provide explainability on the decision process of the model to support its use as a second opinion to oncologists.
(4) We used two datasets, KvasirV1 and Kaither, to evaluate the performance of the model and compared with state-of-the-art (SOTA) methods.

[11] Prabhu, A.J. (2024). Colon cancer detection using vision transformers and explainable AI. International Journal of Advanced Research in Science, Communication and Technology (IJARSCT), 4(1), 189-196.

Comment 2: Incomplete Methodological Transparency Several key components of your methodology are inadequately described. This includes image preprocessing (e.g., patch generation, magnification level), training configuration (e.g., optimizer, learning rate, epochs), and validation design (e.g., test set construction, use of cross-validation). These omissions hinder reproducibility and make it difficult to assess the rigor of your experimental setup.
Response: We thank the reviewer for highlighting the importance of reproducibility and methodological transparency. We acknowledge that certain pieces of information in our initial submission were deficient, and we have already made changes to the text to address these gaps. Specifically:

Image preprocessing and patch generation: We resized images to 300x300x3 for the input to ResNeXt. In the ViT encoder a non-overlapping patch of 16 x 16 is used for the input. Images in the Kaither dataset were histopathology slides with eight tissue classes and a resolution of 150 x 150. Since the dataset was evenly preprocessed, no adjustments to the magnification level were made.

5.5.3. Effect of patch size

This section presents a comparison of the RNTNet model's accuracy on two datasets, Kaither and KvasirV1, with varying patch sizes, i.e., 4×4, 8×8, 16×16, 64×64, and 128×128, as shown in Figure 8. With a precision of 95.17% on the Kaither dataset and 94.27% on the KvasirV1 dataset, the model performed poorly at the smallest patch size of 4×4, which implies that there was insufficient contextual information. RNTNet achieved a precision of 96.05% on Kaither and 95.87% on KvasirV1 when the patch size was increased to 8×8, while the highest precision of 98.20% for Kaither and 97.96% for KvasirV1, was obtained with a patch size of 16×16. Precision dropped when the patch size changed to 64×64. Furthermore, with a patch size of 128×128, precision dropped further to 96.89% and 95.78%, on Kaither and KvasirV1, respectively. These results indicate that the best patch size for achieving the highest classification performance for colorectal cancer detection is 16×16, while patch sizes that are both too small and too large negatively affect the model performance.

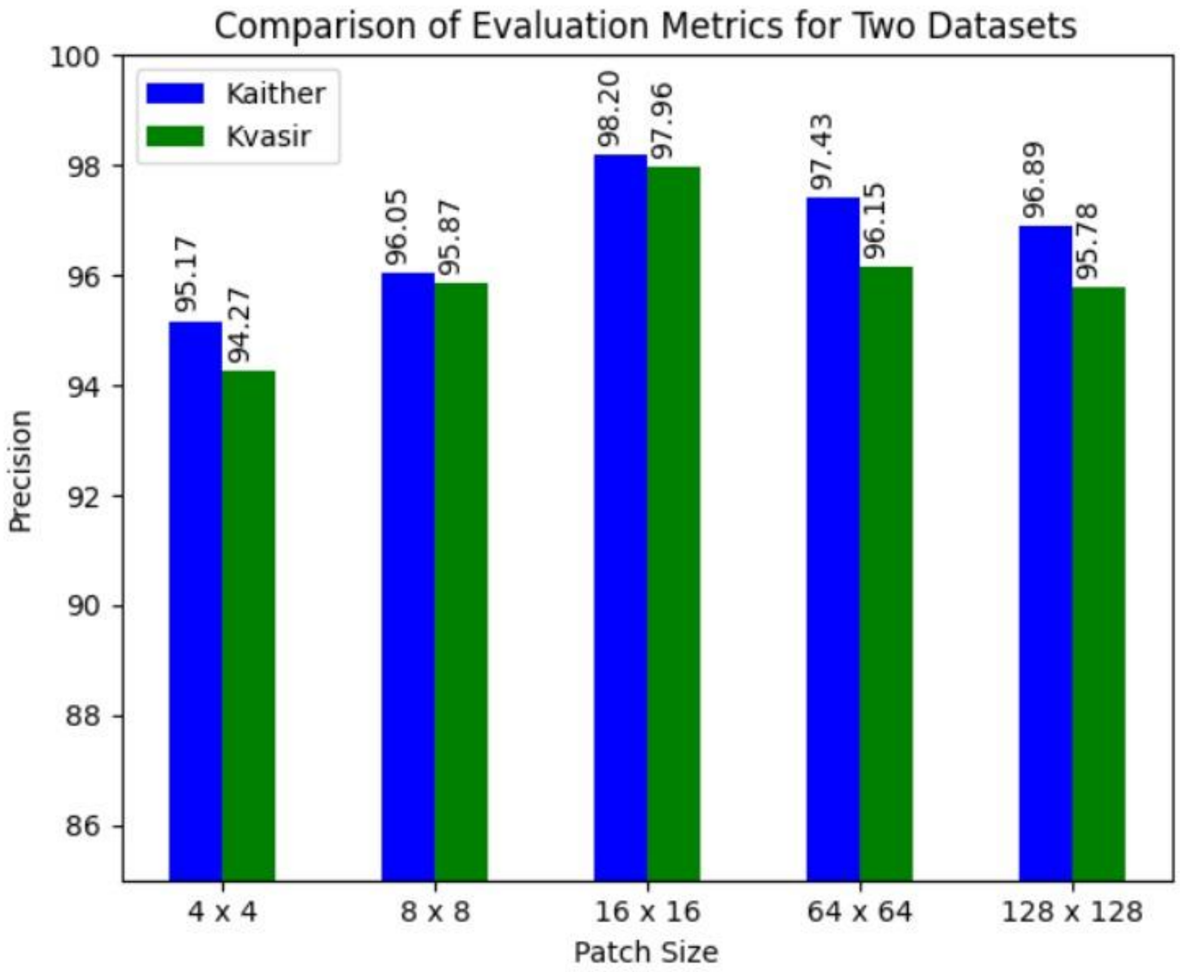

Figure 8. Effect of patch size on Kaither and Kvasir colorectal cancer datasets

Training Configuration: We provided the details of the training configuration in Section 4.2 as follows:

We tested RNTNet with an NVIDIA Quadro RTX-4000 GPU that has 128 GB RAM and an 8GB dual graphics card. Moreover, Python version 3.10 was used for implementation. The ADAM optimizer with a initial learning rate of 0.0001 was employed to train the model for 180 epochs using a batch size of 64. A patch size of 16x16 was used in the ViT encoder to provide local and global attention to the feature map obtained from the ResNeXt.

Validation Design: In section 4.3, we explicitly stated that the dataset was randomly split into 80% for training and 20% for validation. The number of images in each class is equally distributed in both classes. Therefore, we avoid the use of cross-validation to evaluate model performance.

Comment 3: Insufficient Statistical Validation. The manuscript lacks statistical testing to support claims of model superiority. Metrics such as accuracy and F1-score are presented without confidence intervals or hypothesis testing. No standard deviations or cross-validation results are

reported. Without statistical evidence, the performance differences between RTMA and baseline models remain unsubstantiated.

Response: Thank you for your valuable comment. We added Section 5.5.4 Statistical Analysis as follows:

We performed statistical analysis, specifically, a paired t-test, on the performance results presented in Table 6, with the outcomes presented in Figures 9 and 10. Figure 9 shows that the mean difference between precision and Kappa value on the KvasirV1 dataset is 0.44. Further, the p-value is 0.0381, which is much smaller than the statistically significant value of 0.5. Hence, the null hypothesis can be rejected. Moreover, Figure 10 shows a mean difference value of 0.48 on the Kaither dataset. Further, the p-value of 0.00472 <0.5 confirms its statistically significant nature, and the null hypothesis can be rejected.

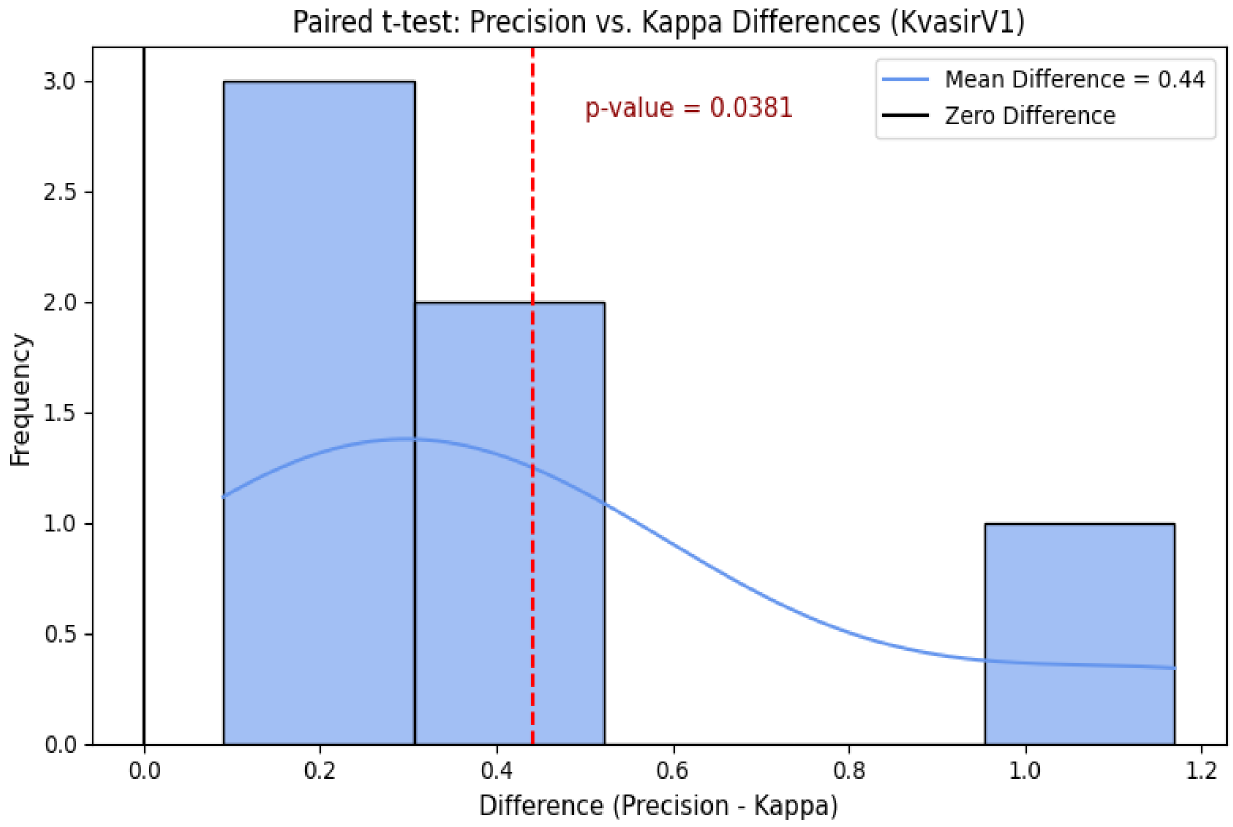

Figure 9. Statistical analysis on the KvasirV1 dataset

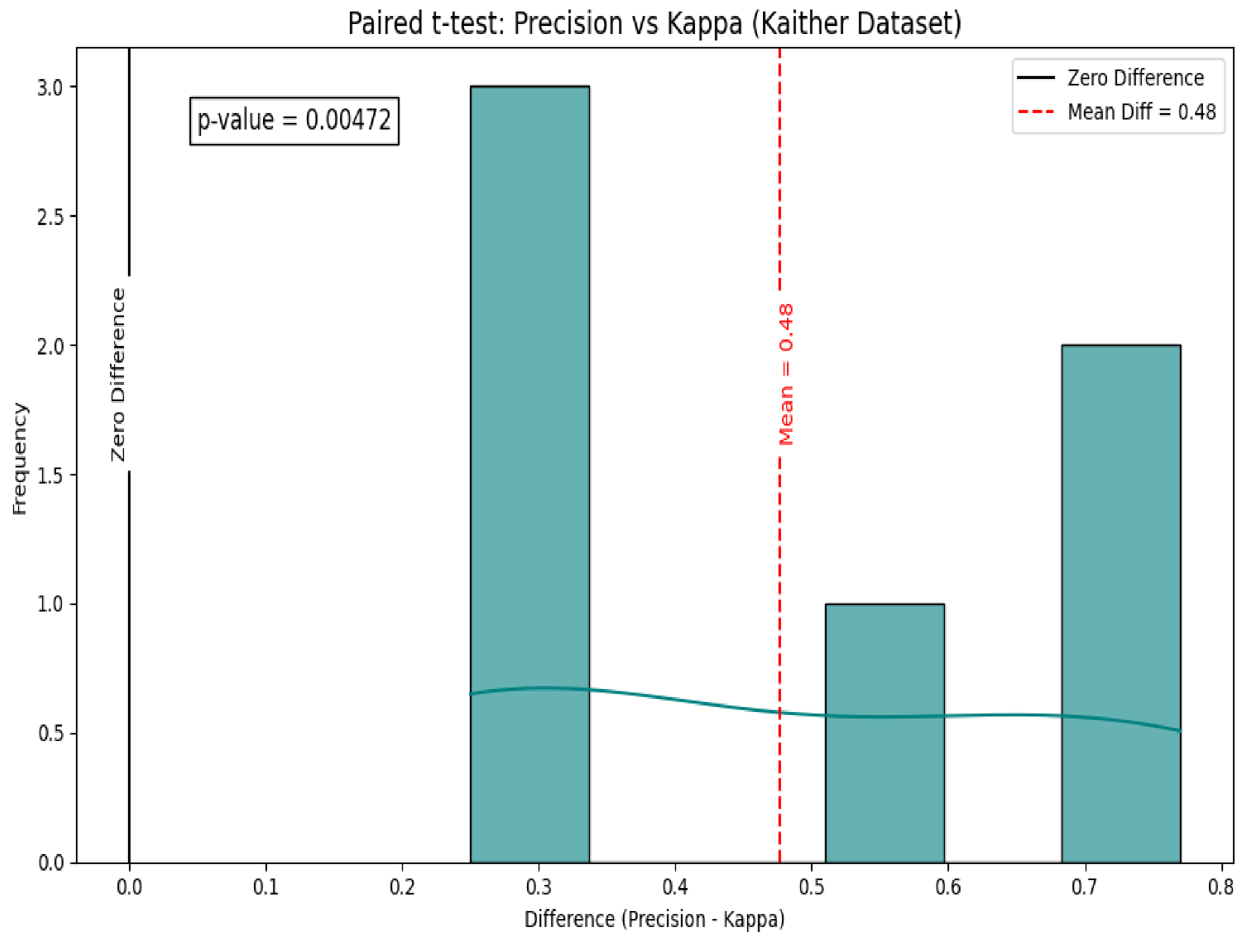

Figure 10. Statistical analysis on the Kaither dataset

Comment 4: Overstated Clinical Claims. The manuscript suggests potential application in "early diagnosis" of colorectal cancer. However, the dataset used (LC25000) is synthetic, lacks patient-level metadata, and is not validated against real-world clinical outcomes. As such, claims about early diagnostic relevance are premature and should be avoided in the absence of clinical data or external validation.

Response: Thank you for this important comment. We utilized two datasets, i.e., Kaither and KvasirV1, which are verified by experts and all annotations are provided by them for disease diagnosis.

The dataset used in the study can be accessed using URL: https://zenodo.org/records/53169 https://datasets.simula.no/kvasir/.

Comment 5: Language and Presentation Issues. While the manuscript is generally understandable, there are numerous grammatical issues, repetitive phrasing, and instances of vague or informal

technical language. These issues negatively impact the readability and professionalism of the work.

Response: Thank you for your valuable suggestion. We performed a thorough review of the manuscript by a native English speaker, and dealt with all grammatical errors, eliminated repetitive phrasing, and replaced informal or unclear technical language with academically appropriate terms. The manuscript has undergone a thorough revision process.

Reviewer #2:

The model architecture is described in details and the analysis is sound. I thank the authors for this solid work. the ViT architecture is super capable in handling image data compared to previous models.

Response: Thank you for your valuable feedback.
