# Supplementary material for: Diagnosis of colorectal cancer using residual transformer with mixed attention and explainable AI
Source: PLoS One. 2025 Nov 3;20(11):e0335418. doi: 10.1371/journal.pone.0335418 (PMC12582454; doi:10.1371/journal.pone.0335418)
Supplement: Attachment — Submitted filename: Reviewer responses.pdf [file pone.0335418.s003.pdf]

Review report

Point-wise Detailed Response to Editor and Reviewers' Comments

Title: Diagnosis of Colorectal Cancer using Residual Transformer with Mixed Attention and Explainable AI

Authors: *Poonam Sharma, Bhisham Sharma, Ajit Noonia, Dhirendra Prasad Yadav, Panos Liatsis*

Dear Editors and Reviewers:

We truly appreciate the time and effort you took to read our manuscript and provide valuable comments. Your comments have been extremely helpful in leading us to enhance and streamline our work. We have carefully considered your suggestions and made the necessary changes in the revised manuscript. We have made every effort to address all the points mentioned and truly hope that the revisions meet your satisfaction. The following are the changes we have made to comply with the received comments.

Reviewer Comments – 1:

Comment 1: Thank you for your responses; however, ResNeXt + ViT hybridization and mixed attention are not conceptually novel. Others have done similar hybrids. What's the actual innovation beyond rearranging known components?

Response: The reviewer's insightful observation is greatly appreciated. We acknowledge that previous studies have been conducted on hybridizing CNNs with transformers and attention mechanisms, such as ResNeXt. However, the conceptual innovation of our work goes beyond simply rearranging familiar components. The principal contributions of the manuscript are as follows.

1. We combine overlapping block attention (OBA) and grid attention (GA) to provide local and global attention to the spatial feature map. In traditional ViTs, multi-head self-attention (MHSA) is applied, which ignores local feature dependencies and has a quadratic computational cost. The necessity and efficacy of this design are demonstrated by the ablation study (Table 6), which shows that OBA and GA together significantly outperform MHSA.

2. Although there are ResNeXt + ViT hybrids, they usually employ standard MHSA, which is computationally costly O (M^4D). Our suggested mixed attention block provides an accurate yet computationally efficient solution by reducing complexity to O (M^2D^2). The complexity analysis (Table 7) demonstrates how RNTNet achieves superior accuracy while achieving a better balance between accuracy and efficiency than more complex models like YOLOv9 and MaxViT.
3. Our model is validated on two different and clinically relevant imaging modalities, including endoscopic (KvasirV1) and histopathology images (Kaither).
4. To enhance interpretability and facilitate second opinions, we have added Grad-CAM visualizations alongside performance metrics. This integration sets our method apart from earlier work, which primarily concentrated on accuracy, and is crucial for clinical adoption.

Comment 2: State the actual slide magnification (or explicitly say if that info wasn't available). For example, "all slides were provided at 40× magnification."

Response: We value the reviewer's recommendation. We have now specified the slide magnification in the updated manuscript. The Kaither NCT-CRC-HE-100K dataset comprises 224 × 224-pixel hematoxylin and eosin (H&E) stained histopathology patches extracted from whole-slide images digitized at a resolution of 0.5 μm per pixel, corresponding to approximately 20× optical magnification.

Comment 3: Trim hardware specs down to just GPU model + VRAM. The kind of detail you wrote isn't as important for reproducibility as batch size, optimizer, etc.

Response: We are grateful for the reviewer's recommendation. Only the GPU model and VRAM, two crucial pieces of hardware, are now documented in the experimental settings section. The important training parameters, such as batch size, optimizer, and learning rate, which are more pertinent to reproducibility, have been retained, and the rest have been removed.

Comment 4: You wrote "statistically significant value of 0.5." That's almost certainly a typo for 0.05. Using 0.5 makes it look like you don't understand significance testing.

Response: We appreciate the reviewer bringing this error to our attention. In fact, the previous reference to 0.5 was a typographical error; the proper threshold for statistical significance is 0.05. To prevent any misunderstandings, we have fixed this throughout the manuscript.

Comment 5: Report 95% confidence intervals for key metrics (accuracy, F1, precision, recall). Bootstrapping over test samples is fine if you didn't use cross-validation.

Response: Thank you for your concern. As suggested, we applied a 5-fold cross-validation scheme on the KvasirV1 and Kaither datasets and presented them in Tables 8 and 9 in the revised manuscript.

Comment 6: Clarify how the t-test was conducted (e.g., "we compared per-class accuracies across the eight classes" or "we ran the model 5 times with different seeds and compared runs").

Response: We appreciate the reviewer's feedback regarding the clarity of the statistical test procedure. In the revised manuscript, we have provided a clear explanation of how the paired t-test was conducted. Specifically, we employed the paired t-test for per-class performance indicators (precision and Cohen's kappa) on the eight classes of the KvasirV1 and Kaither datasets. The model's predicted outcomes for every class were compared to the ground truth to obtain the per-class metric values. These values were then paired and analyzed using a two-tailed paired t-test to determine if the differences between measures (such as accuracy vs. kappa) were statistically significant.

This approach was selected to ensure that the statistical test reflected both class-wise variability and aggregate performance. As made clear in the updated Results section (Statistical Analysis, Section 5.5.4), the paired t-test was conducted on per-class performance values (precision and kappa) across the eight categories of each dataset. The class-wise scores were compared and paired to see if the observed differences were statistically significant. The associated figures (Figures 9 and 10) show these results along with the mean differences and p-values.

Comment 7: If you cannot add cross-validation, explicitly state the reason and acknowledge it as a limitation.

Response: We appreciate the reviewer highlighting the value of cross-validation. We applied a 5-fold cross-validation scheme to train and test the model's performance on the Kaither and KvasirV1 datasets. The quantitative results are depicted in Tables 8 and 9.

Table 8 displays the performance of the proposed model on the KvasirV1 dataset using 5-fold cross-validation. In Fold 1, the model showed consistent initial performance, attaining 95.18% accuracy with a precision of 94.30%, a recall of 93.89%, and an F1-score of 94.07%. Fold 2

demonstrates an accuracy of 96.40%, a recall of 94.29%, and a precision of 95.06%. Fold 3 further enhanced performance with a 96.87% accuracy. Fold 4 achieved an accuracy of 97.08% and an F1-score of 96.41%. The highest performance is observed in Fold5, with an accuracy of 98.67%, F1-score of 97.40% and Kappa of 97.14%. Overall, the average performance of all five folds is 96.80% accuracy, 95.90% precision, 95.43% recall, 95.57% F1-score, and 95.29% kappa. The results show that the proposed method performs consistently and reduces the risk of overfitting to the KvasirV1 dataset.

Table 8. Performance of the proposed model on the KvasirV1 dataset using 5-fold cross-validation

Folds	Accuracy (%)	Precision (%)	Recall (%)	F1-score (%)	Kappa (%)
Fold_1	95.18	94.30	93.89	94.07	93.48
Fold_2	96.40	95.06	94.29	94.97	94.16
Fold_3	96.87	96.10	95.60	95.90	95.48
Fold_4	97.08	96.57	96.08	96.41	96.17
Fold_5	98.67	97.46	97.30	97.40	97.14
**Average**	**96.80**	**95.90**	**95.43**	**95.75**	**95.29**

The performance of the proposed model on the Kaither dataset across all five cross-validation folds is shown in Table 9. The model's accuracy in Fold 1 was 96.06% with 95.81% precision. Fold 2 showed improvement with 97.43% accuracy, 96.28% precision, and 95.86%. Further, the Fold 3 model achieved an accuracy and precision value of 98.13% and 97.09%, respectively. Fold 4 showed steady generalization with 98.50% accuracy, 97.52% precision, 97.12% recall, 97.37% F1-score, and 97.03% Kappa. The model obtained 97.49% Kappa, 98.17% recall in Fold 5. Moreover, the model's average precision and Kappa score are 96.98% and 96.17%, respectively.

Table 9. Performance of the proposed model on the Kaither dataset using 5-fold cross-validation

Folds	Accuracy (%)	Precision (%)	Recall (%)	F1-score (%)	Kappa(%)
Fold_1	96.06	95.81	94.79	95.33	94.31
Fold_2	97.43	96.28	95.86	96.22	95.45
Fold_3	98.13	97.09	96.73	96.91	96.54

Fold_4	98.50	97.52	97.12	97.37	97.03
Fold_5	98.67	98.20	98.17	98.11	97.49
**Average**	**97.76**	**96.98**	**96.53**	**96.79**	**96.17**

Comment 8: You didn't actually address what I asked. My concern was that your overstated clinical claims about "early diagnosis" without clinical-level validation. your answer only tells me that your datasets are expert-annotated and drops some URLs. That doesn't solve the problem; in fact, it feels like a deflection. The key issues (synthetic LC25000, lack of patient metadata, and no outcome validation) are still unacknowledged. If you leave "early diagnosis" phrasing in the paper, I will still consider it misleading. Providing links to Kaither and KvasirV1 is nice for transparency, but it doesn't change the fact that these datasets are benchmarks, not clinical datasets. your tone also feels a little dismissive. Instead of conceding that your clinical claims were too strong, you doubled down by emphasizing "verified by experts." That's not the same thing as real-world clinical validation.

Response: We sincerely thank the reviewer for this important insight. We acknowledge that our initial use of the term "early diagnosis" was very strong and could have been interpreted as implying direct clinical validation, which is not supported by the way our current study was designed. Although the Kaither and KvasirV1 datasets are annotated benchmark datasets, as the reviewer correctly points out, they do not have longitudinal outcomes, patient-level metadata, or prospective validation in real-world clinical settings. Furthermore, we did not utilize artificial datasets such as LC25000 in our research, but we completely agree that without clinical metadata and validation, the results cannot yet be applied to patient care or real-world diagnostic deployment. We modified the "early diagnosis" term to diagnosis in the revised manuscript.

In the limitations section, we have made it clear that our results are based solely on benchmark datasets and that before any claims of diagnostic value can be made, clinical-level validation on patient cohorts, including outcomes and metadata, would be necessary.

Thanks, and Regards
Authors.
